# A Review on Medicinal Plants Used in the Management of Headache in Africa

**DOI:** 10.3390/plants10102038

**Published:** 2021-09-28

**Authors:** Ebenezer Kwabena Frimpong, John Awungnjia Asong, Adeyemi Oladapo Aremu

**Affiliations:** 1 Indigenous Knowledge Systems Centre, Faculty of Natural and Agricultural Sciences, North-West University, Private Bag X2046, Mmabatho 2790, South Africa; johnmilan058@gmail.com; 2 Food Security and Safety Niche Area, Faculty of Natural and Agricultural Sciences, North-West University, Private Bag X2046, Mmabatho 2790, South Africa

**Keywords:** asteraceae, Fabaceae, traditional medicine, orthodox conventional medicine, indigenous population

## Abstract

The use of medicinal plants in the management of diverse ailments is entrenched in the culture of indigenous people in African communities. This review provides a critical appraisal of the ethnobotanical uses of medicinal plants for the management of headache in Africa. Research articles published from 2010 (Jan) to 2021 (July) with keywords such as Africa, ethnobotany, headache, medicinal plant and traditional medicine were assessed for eligibility based on sets of pre-defined criteria. A total of 117 plants, representing 56 families, were documented from the 87 eligible studies. Asteraceae (10%), Fabaceae (10%), Lamiaceae (9%) and Mimosaceae (5%) were the most represented plant families. The most popular plant species used in the management of headache were *Ocimum gratissimum* L. (*n* = 7), *Allium sativum* L. (*n* = 3), *Ricinus communis* L. (*n* = 3) and *Artemisia afra* Jack. ex. Wild (*n* = 2). The leaves (49%), roots (20%) and bark (12%) were the most common plant parts used. Decoction (40%) and infusion (16%) were the preferred methods of preparation, whereas the oral route (52%) was the most preferred route of administration. The data revealed that medicinal plants continue to play vital roles in the management of headache in African communities. In an attempt to fully explore the benefits from the therapeutic potential of indigenous flora for common ailments, further studies are essential to generate empirical evidence on their efficacies, using appropriate test systems/models. This approach may assist with the ongoing drive towards the integration of African traditional medicine within mainstream healthcare systems.

## 1. Introduction

From time immemorial, humans have relied on medicinal plants to fight diseases and provide for diverse health needs. According to the World Health Organisation (WHO), about 80% of the world’s population relies on medicinal plants for their primary health care needs [1]. In Africa, many people depend on medicinal plants to fight ailments, including headache. Headache is a common health condition in both children and adults [2]. Primary headache has no fundamental cause, whereas secondary headache is caused by conditions such as brain tumor, neck injury and sinus infection [3]. The pain experienced during headache emanates from a combination of signals in the brain, blood vessels and proximate nerves of an individual. Precise nerves in an individual’s blood vessels and the muscles in the head switch on, which send pain signals to the brain [4]. “The common causes of headache are illness (fever, cold and infections), environment noise, stress, pollution, strong smell from perfumes or household chemicals), genetics (children whose parents had migraine headaches history tend to have them in their lifetime)” [5]. Headache accounts for 5% of the worldwide disease burden in terms of disability [6]. The prevalence rate varies across different countries, such as 3% in Ethiopia [7], 52.2% in Turkey [8] and 92.4% in Pakistan [9]. Relative to the low prevalence rate recorded in Ethiopia [7], a higher rate was evident in Turkey and Pakistan, which may be attributed to the presence of heavy industries (major sources of atmospheric pollution). The Ethiopian study was conducted in a rural area (Meskan and Marko) [7] compared to the urban areas (study sites) in Turkey (Mersin) [8] and Pakistan (Karachi) [9].

Generally, the management of headache in orthodox conventional medicine (OCM) involves the use of analgesics, non-steroid anti-inflammatory drugs (NSAIDs), *beta*-blockers, calcium channel blockers, anticonvulsants and tricyclic antidepressants [10]. As highlighted in Table 1, these drugs are associated with a diverse range of side effects, which continue to stimulate the search for alternative treatment regimens from different sources, especially higher plants. As an indication of the potential of medicinal plants for managing headache, 96 plants were identified as local remedies for headache among three tribes (Zulu, Xhosa and Sotho) in South Africa [11]. Studies have highlighted the potential of medicinal plants in the management of headache in many African countries. A study conducted in Ghana revealed the use of medicinal plants such as *Griffonia simplicifolia* (DC.) Baill. by the indigenous population in the management of headache [12]. Similarly, *Cinnamosma fragrans* Baill. was used by the indigenous population in Madagascar for the management of headaches [13]. Given the high reliance on traditional medicine, it is pertinent to identify the common medicinal plants employed to manage headache by the indigenous population across African communities. This will assist with the scientific identification and evaluation of the medicinal plants with the potential to mitigate the effects of headache. Thus, the present review provides an inventory and critical appraisal of medicinal plants used for the management of headache by different tribes in Africa.

## 2. Methods

### 2.1. Selection of Published Articles

Google Scholar, Pub-med, the web of science and the Cochrane Library electronic databases were searched to identify potential research articles. Medicinal plants used for the relief of headache in Africa, traditional medicine (TM), TM and headache in Africa and the pathophysiology of headache were a few keywords and phrases used to identify eligible articles.

### 2.2. Selection Criteria

**Figure 1:**
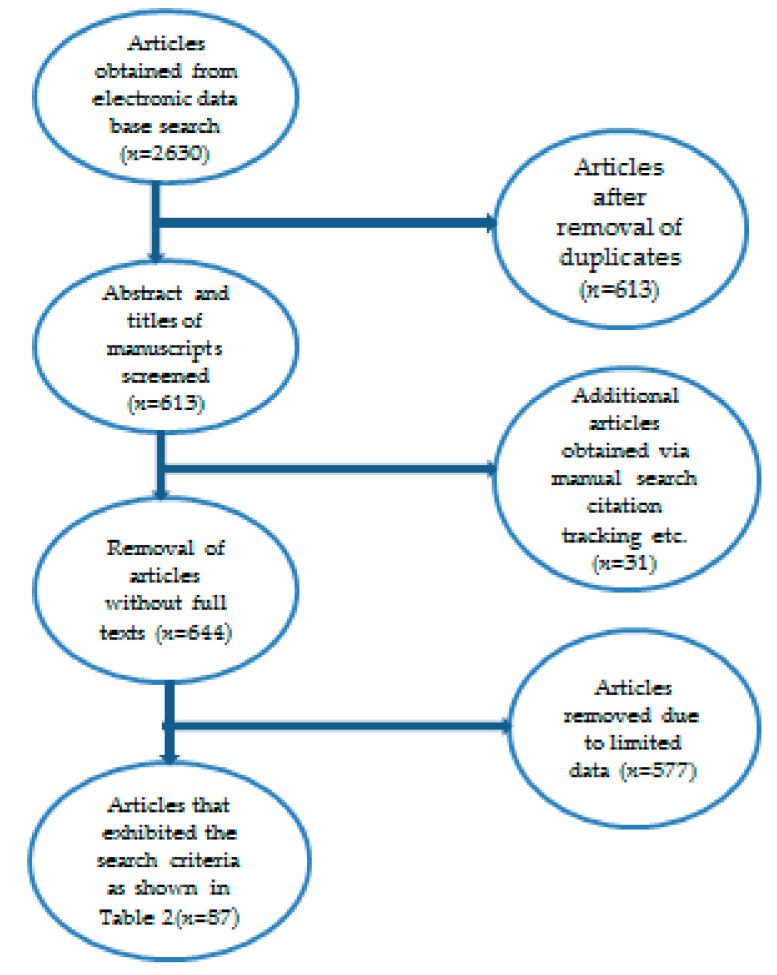
An overview of the procedure applied for the identification of 87 articles included in this review.

The main inclusion criterion was published research articles related to the use of plants for managing headache, with duration from January 2010–July 2021. The scientific name of the plant had to be provided and the study area had to be located in an African country. The exclusion criteria were published articles not written in the English language. Moreover, review articles (literature or systematic), studies involving the use of OCM in the management of headache, abstract-only accessed published articles, *in vivo*/*in vitro* studies, letters, case reports, books, manuals and all those reporting animal/mineral-based TM used in the management of headache were excluded. Generally, four-step selection criteria were employed to identify the published articles included in this study. Firstly, the importance of studies was checked based on the captions of the published articles. Secondly, abstracts of the published articles were assessed to match the inclusion criteria. Thirdly, the full-length text of the identified published articles based on the knowledge acquired in step 2 was obtained and read thoroughly in order for the authors to make an informed decision on whether to include/reject those research articles in this review. Published articles that met the inclusion criteria were retrieved for careful evaluation. In total, 87 published articles were included in this study (Figure 1). The data were mined to extract information such as botanical names, local names, countries, routes of administration and modes of preparation (Table 2). The scientific names for the plants were validated using recognised databases including The Plant List (including Global Biodiversity information facility) and Plant ZA [29,30].

## 3. Results and Discussion

### 3.1. Pattern and Distribution of Medicinal Plants Used in the Management of Headache in Africa

The 87 eligible studies included in this study were obtained from 27 African countries (Table 2). The countries that recorded the highest number of plant species were as follows: South Africa (*n* = 30), Nigeria (*n* = 16), Ethiopia (*n* = 13) and Uganda (*n* = 12). In total, 117 medicinal plants, representing 56 families, were documented in this review. Irrespective of the geographical location in Africa, the use of medicinal plants in mitigating the effects of headache is of great prevalence (Table 2). This is in agreement with similar studies conducted in Asia and Europe, which revealed the use of medicinal plants by the general population in the management of headache [31,32].

### 3.2. Overview of Medicinal Plants and Families Used in the Management of Headaches

**Figure 2:**
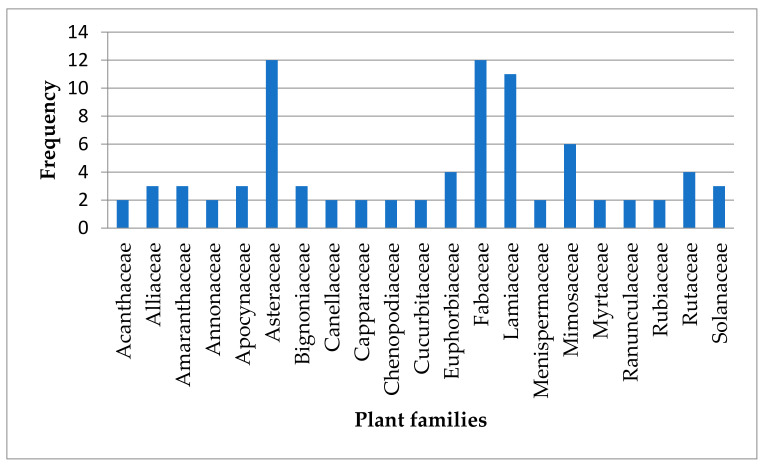
Frequency (*n*) of plant families mentioned (*n* ≥ 2) as remedy for managing headache in Africa. The remaining plant families (*n* = 34) that were mentioned once are listed in Table 2.

In this study, most of the identified medicinal plants belonged to Asteraceae (10%), Fabaceae (10%) Lamiaceae (9%) and Mimosaceae (5%) (Figure 2). According to van Wyk [118], African Traditional Medicine in sub-Saharan African is dominated by plant families such as Lamiaceae (142 spp., 37 genera), Asteraceae (314 spp., 112 genera) and Fabaceae (567 spp., 156 genera). This observation is comparable to the dominant plant families (Lamiaceae, Asteraceae, Fabaceae) associated with the identified medicinal plants used for managing headache in Africa. The selection of medicinal plants (for some families) used in the management of diseases in sub-Saharan Africa could be influenced by culture and the availability of a plant species in a geographical location [118]. Significantly, in comparison with studies conducted in different parts of the globe to ascertain the medicinal plants used in the management of headache, most of the identified medicinal plants were members of Lamiaceae and Asteraceae. For instance, in Iran, Lamiaceae and Asteraceae were the most frequently cited plant families used for managing headache [119]. Likewise, in Serbia, the aerial parts of the identified plant species *Teucrium montanum* L. and *Mentha pulegium* L. used in the management of headache belonged to the Lamiaceae [120].

Certain plant species associated with the most cited plant families (Lamiaceae, Asteraceae, Fabaceae and Euphorbiaceae) were frequently mentioned in this study. *Ocimum* *gratissimum* L. (*n* = 7), belonging to Lamiaceae, which was frequently mentioned in this study, is known to possess diverse pharmacological activities, such as antioxidant and anti-inflammatory properties [121]. Similarly, *Artemisia afra* Jack. ex. Wild (*n* = 2), belonging to the Asteraceae and one of the most cited plant species, possesses analgesic, anti-inflammatory and antidepressant pharmacological activities [122]. Furthermore, *Ricinus communis* L. (*n* = 3), which is a member of Euphorbiaceae, is associated with anti-oxidant and anti-inflammatory properties [123].

### 3.3. Commonalities in the Use of Similar Medicinal Plants in the Management of Headache in Africa

The findings of the current review identified some similarities that exist in the use of particular medicinal plants in the management of headache among some African countries. For example, the use of *Artemisia afra* Jack. ex. Wild was reported in South Africa [35] and Ethiopia [58]. Furthermore, a study conducted in South Africa revealed the analgesic effects of *Artemisia afra* Jack. ex. Wild, which may attenuate pain associated with headache. The experimental results confirmed the increase in the reaction time of Wister rats during a hot plate test when the animals were administered with extracts of *Artemisia afra* Jack. ex. Wild [124]. Similarly, *Ocimum gratissimum* L., a plant cultivated in both Ghana [125] and Madagascar [13] and used by local inhabitants to manage headache, reduced the pain response of albino rats considerably during a hot plate experimental study [126]. These examples justify the traditional use of the medicinal plants in the treatment and management of headache. However, issues about the safety and efficacy of medicinal plants used by the indigenous population in traditional African communities to mitigate ailments are of great concern to the WHO [127], especially in cases of adverse responses associated with the use of medicinal plants by patients. For example, a medical case report study indicated that the ingestion of *Nicotiana glauca* Graham, belonging to the same genus as *Nicotiana tabacum* L., which is used in Cameroon to treat headache [43], caused serious respiratory problems for a 60-year-old healthy patient in Greece [128]. This highlights the need for the standardisation of herbal medicine as it is critical to ensure its safety and efficacy [127].

### 3.4. Plant Parts Used to Manage Headache in Africa

**Figure 3:**
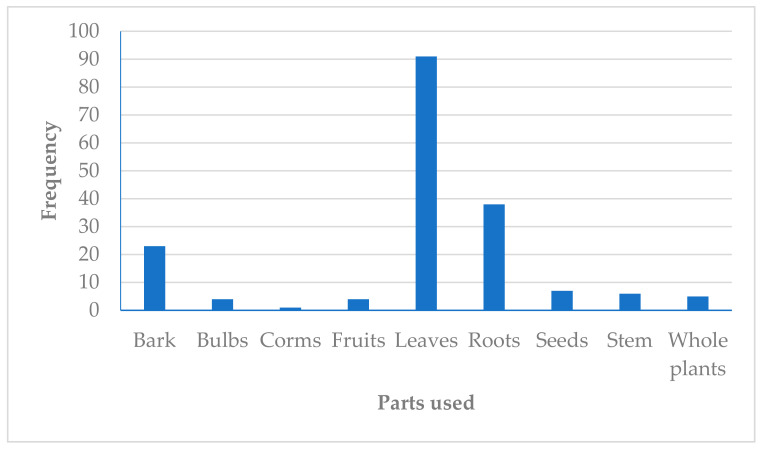
Frequency (*n*) of medicinal plant parts used as herbal preparations for managing headache in Africa.

The leaves (49%), root (20%) and bark (12%) were the dominant plant parts used in the management of headache (Figure 3). This is in agreement with studies conducted in Iran, where the usage of these plant parts (leaves, root and bark) for the management of headache has been documented [45]. The popularity of leaves as the most preferred plant part in the preparation of herbal mixtures by traditional health practitioners (THPs) established in many ethnobotanical studies could be due to the fact that leaves are more accessible. Even though the roots are rich in phytochemicals, their usage for the preparation of herbal mixtures is sometimes restricted because their frequent usage could pose a potential risk to the survival of the plants [46].

### 3.5. Method of Preparation of Medicinal Plants Used in the Management of Headache in Africa

**Figure 4:**
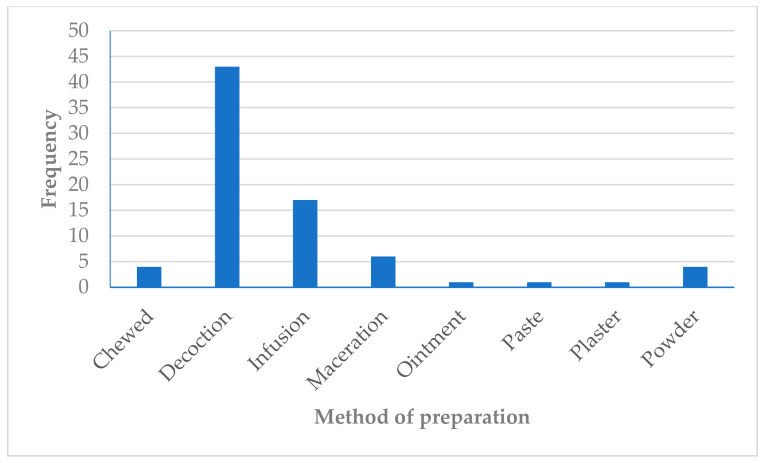
Frequency (*n*) for the method of preparation of medicinal plants used in the management of headache in Africa.

In the included studies, the most common methods of preparation of the medicinal plants were decoction and infusion (Figure 4). Decoction and infusion involve the boiling of different parts of the medicinal plant to extract its bio-active compounds to be administered to patients to mitigate the effects of certain ailments. Decoctions (40%) and infusions (16%) are generally easy to prepare and inexpensive, which is why they are predominantly employed by THPs in the management of diseases [129]. In the Philippines and Pakistan, decoctions and infusions were the preferred methods used for the preparation of medicinal plants [130,131]. Likewise, in similar reviews of ethnobotanical studies about the management of diseases on the African continent, the constituents of herbal mixtures prepared as decoctions and infusions by THPs are rarely mentioned [132,133]. This shortcoming deters the efforts of health departments and policy makers to recommend the use of such herbal mixtures in mainstream healthcare. Thus, the scientific evaluation of herbal mixtures could be instrumental in the development of novel pharmaceuticals for the management of diseases across the continent. To attain such an important goal, collaboration among stakeholders (THPs, researchers and relevant government departments) is needed across the continent. The recognition of the intellectual property of THPs and benefits thereof will be instrumental for collaboration and solving the dilemma of withholding knowledge by THPs.

### 3.6. Route of Administration of Medicinal Plants Used in the Management of Headache in Africa

**Figure 5:**
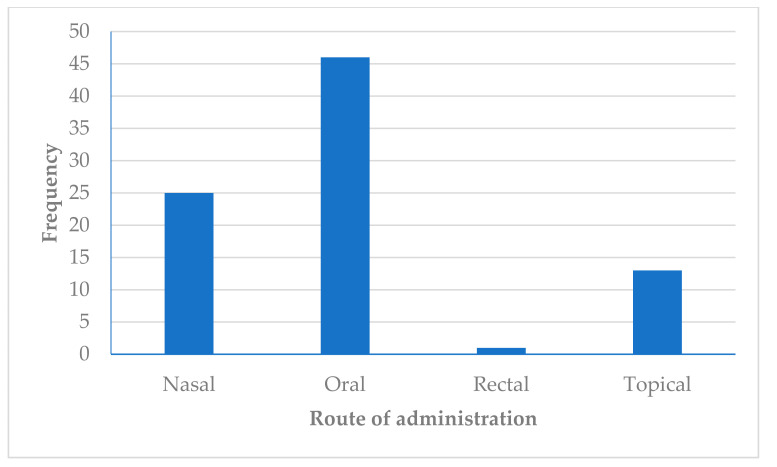
Frequency (*n*) for the route of administration of herbal mixtures used in the management of headache in Africa.

The route of administration of decoctions, infusions and boiled samples of the medicinal plants was mainly (52%) oral (Figure 5). These findings corroborate those of previous studies [51,52]. The oral route of administration is mostly preferred because it is an effective, non-invasive and suitable method of administration [53]. In the current study, other routes of administration of the burnt/dried medicinal plants included inhalation/sniffing. Interestingly, the inhalation of burnt and dried roots of *Calotropis gigantea* was used for the treatment of headache in Gujarat, India [54]. Though not reported in the articles reviewed for this study, inhalation has been shown to be effective as it conveys the drug or active ingredient to the target organ with a reduction in systemic side effects [134]. The topical application of medicinal plants was the third most commonly cited route of administration used in the management of headache (Figure 5). This is in congruent with studies conducted in other countries. For instance, the topical application of medicinal plants, such as *Alpinia galanga* Sw. (rhizome) and *Baccharis latifolia* (Ruiz and Pav.) Pers. (leaves and bark), were used in alleviating the effect of headache in patients in India [135] and Peru [136], respectively.

## 4. Conclusions

This review focused on the use of medicinal plants in the treatment and management of headache on the African continent. A total of 117 medicinal plants were documented from the 87 eligible studies included in this study. The most popular plant species used in the management of headache were *Ocimum gratissimum*, *Allium sativum*, *Ricinus communis* and *Artemisia afra*. The findings from this study revealed, to a certain extent, the holism of traditional health practices across the African continent, given that similar plants, methods of preparation and routes of administration were evident. This interesting observation can assist researchers in the field of TM to design a uniform health protocol(s) to be used by THPs in the management of headache on the continent. This review provided baseline data for scientific considerations in the quest for natural-based drug development for the treatment of headache. Besides, the methodological documentation of medicinal plants which is supported by scientific data is critical for their assimilation into the primary health care system, which many people rely on due to its affordability and perceived safety compared to OCM [137]. However, there is a need to evaluate the efficacy and contra-indications of herbal preparations, and to establish an accepted dosage and protocol(s) for THPs across the continent to be administered to patients. This is important because herbal medicines have the possibility of increasing or decreasing the pharmacological action of OCM in older people and people with weaker immune systems [138]. Thus, scientists need to establish the interactions between herbal mixtures and OCM, which may assist in saving lives.

## Figures and Tables

**Table 1:** Orthodox conventional medicines used in the management of headache.

Classification	Drugs and Their Synonyms	Mechanism of Action	Pharmacological Effects	Side Effects
Analgesics	Analgin Paracetamol	Reduction of the pain impulses via afferent nerves and inhibition of subcortical pain centres [14,15] Reduction of the pain impulses via afferent nerves and inhibition of subcortical pain centres [15].	Analgesic and antipyretic	Nausea and vomiting, dizziness and skin rash [14]. Skin rash, sore throat, unusual tiredness or weakness, liver damage [16].
Non-steroid Anti-inflammatory drugs	Aspirin Indomethacin	Overpowering the cyclooxygenase activity (COX), subdual of the inflammatory mediators. The reticence of the subcortical pain centres [17].	Anti-inflammatory, antipyretic and analgesic	Stomach or gut irritation, indigestion and nausea [18]. Gastric ulcerations, allergic reactions [19].
*Beta*-blockers	Propranolol Nadolol Metropol Atenolol	*Beta*-blockers help in the reduction of blood flow in the brain by directly acting on the blood vessels in the organ. Significant widening of blood vessels, known as vasodilation, is associated with migraine [20].	Antihypertensive	Fatigue, dizziness, poor blood circulation and sexual dysfunction in males [21].
Calcium channel blockers	Verapamil	Helps in the inhibition of Ca^2+^ ion transport inside the smooth muscles’ vessels. This helps in the prevention of the mechanical tightening of the muscle wall of the artery [22].	A decrease in blood pressure	Dizziness, constipation, stomach upset [23].
Anticonvulsants	Sodium valproate Topiramate	Helps in the increment of the content of inhibitory transmitter gamma-aminobutyric acid (GABA) in the central nervous system (CNS) [24].	Anticonvulsants	Stomach pain, diarrhoea, dry or sore mouth [25]. Tiredness, drowsiness, dizziness [26].
Tricyclic antidepressants	Amitriptyline Venlafaxine	Helps in the reduction of noradrenaline, dopamine and serotonin reuptake thereby increasing their accrual in the synaptic cleft [27].	Antidepressants	Dry mouth, headache. weight gain Dizziness, headache and constipation, sexual dysfunction [28].

**Table 2:** Overview of the medicinal plants used in the management of headache in Africa.

Botanical Name (Family)	Country	# Local Name(s)	Part(s) Used	Method(s) of Preparation	Route of Administration	Reference(s)
*1 Acacia ataxacantha* DC. (Fabaceae)	Namibia	Mukoro (NS)	Not reported	Not reported	Not reported	[33]
*2 Acacia brevispica* Harms (Mimosaceae)	Kenya	Mukuswi (Ka)	Leaves	Crushed	Topical	[34]
*3 Acacia karroo* Hayne (Fabaceae)	South Africa	Umnga (X)	Leaves	Infusion	Oral	[35]
*4 Acacia macrostachya* DC. (Mimosaceae)	Burkina Faso	Sinsindinga (Mo)	Fruit	Decoction	Not reported	[36]
*5 Acacia nilotica* (L.) Delile (Fabaceae)	South Africa	Mugamazu (NS)	Root	Decoction	Not reported	[37]
*6 Acacia oerfota* (Forssk.) Schweinf. (Mimosaceae)	Sudan	Laot (NS)	Root	Burnt	Nasal (inhaled)	[38]
*7 Acacia pennata* (L.) Wild. (Mimosaceae)	Burkina Faso	Kanre (Mo)	Leaves	Decoction	Not reported	[36]
*8 Acacia rehmanniana* Schinz (Fabaceae)	South Africa	Mosemane (Xi, S)	Leaves	Burnt	Nasal (inhaled)	[39]
*9 Aerva lanata* (L.) Juss. (Acanthaceae)	Uganda	Mwenza (NS)	Leaves	Infusion	Not reported	[40]
*10 Pericopsis laxiflora* (Baker) Meeuwen) (Fabaceae)	Cote d’Ivoire	Kolokolo (NS)	Leaves, stem, bark, root	Decoction, maceration, infusion	Oral	[41]
*11 Agapanthus africanus* (L.) Hoffmanns. (Agavaceae)	South Africa	Not reported	Root, leaves	Infusion	Oral	[42]
*12 Ageratum conyzoides* (L.) L. (Asteraceae)	Cameroon	Libolikana (mbo) Ewuda nyo na nyo (NS)	Leaves	Decoction	Not reported	[43]
*13 Albizia amara* (Roxb.) B. Boivin (Mimosaceae)	Kenya	Olperr-elongo (Ma)	Leaves, bark	Not reported	Not reported	[44]
*14 Allium cepa* L. (Alliaceae)	Nigeria	Alubosa (Y)	Leaves, root	Not reported	Topical	[45]
	Chad	Bassalaye (CA)	Bulb	Decoction	Oral	[46]
*15 Allium sativum* L. (Alliaceae)	Nigeria	Nechshinkrut (NS)	Bulb	Crushed	Oral	[47]
	Ethiopia	Nechshinkurte (Am)	Bulb	Chewed	Oral	[48]
	Ethiopia	Sika (G)	Leaves	Chewed	Oral	[49]
*16 Alternanthera sessilis* (L.) R. Br.ex DC. (Amaranthaceae)	Nigeria	Not reported	Whole plant	Not reported	Not reported	[50]
*17 Alternanthera pungens* Kunth (Amaranthaceae)	Ghana	Nkasee nkasee (T)	leaves	Crushed	Rectal	[51]
*18 Andrachne ovalis* (E.Mey.ex Sond.) Mull.Arg. (Phyllanthaceae)	South Africa	Umbeza (Z)	Root	Burnt	Nasal (sniffed)	[52]
*19 Anthonotha macrophylla* P Beauv. (Fabaceae)	Liberia	Not reported	Leaves	Not reported	Not reported	[53]
*20 Aristolochia bracteolatabracteolate* Lam. (Aristolochiaceae)	Sudan	Um galagil (NS)	Aerial part	Infusion	Not reported	[54]
*21 Artemisia absinthium* L. (Asteraceae)	Ethiopia	Dunko (Ma), Duno (Ar)	Leaves, stem	Not reported	Oral, nasal (inhaled)	[55]
*22 Artemisia abyssinica* Sch.Bip. ex A. Rich. (Asteraceae)	Ethiopia	Fara agupiya (NS)	Leaves	Crushed	Oral, nasal	[56]
*23 Artemisia afra* Jack.ex.Wild (Asteraceae)	South Africa	Mhlonyane (Z)	Leaves	Infusion	Not reported	[57]
	Ethiopia	Chikun (AO)	Leaves	Chewed	Oral	[58]
*24 Aspalathus linearis* (Burm.f.) R. Dahlgren (Fabaceae)	South Africa	Rooibos(X), inkanga(X)	Leaves	Decoction	Oral	[35]
*25 Asparagus plumosis* Baker (Asparagaceae)	Mozambique	Munhassuru (CT, CN)	Leaves	Decoction	Nasal (inhaled)	[59]
*26 Aspilia africanaAfricana* (Pers.) C.D. Adams (Asteraceae)	Nigeria	Not reported	Leaves, root	Decoction	Topical	[60]
*27 Azadirachta indica* A. Juss. (Meliaceae)	Nigeria	Not reported	Leaves, bark	Decoction	Oral	[61]
*28 Balanites aegyptiaca* (L.) Delile (Zygophyllaceae)	Kenya	Olgoswa (NS)	Bark	Decoction	Oral	[62]
	Kenya	Kilului (Ka)	Fruit	Infusion	Oral	[63]
*29 Boscia salicifolia* Oliv. (Capparaceae)	Uganda	Ror (NS)	Root	Decoction	Not reported	[40]
	Tanzania	Muguluka (NS)	Root, bark	Not reported	Oral	[64]
*30 Brugmansia candida* Pers. (Solanaceae)	Madagascar	Detora (NS)	Leaves	Not reported	Not reported	[65]
*31 Bryophyllum pinnatum* (Lam.) Oken. (Crassulaceae)	Cameroon	Yoka (oroko) Elualua (Bakweri) (NS)	Leaves, root	Maceration	Not reported	[43]
*32 Buchholzia coriacea* Engl. (Capparaceae)	Nigeria	Not reported	Bark	Crushed	Nasal (inhaled)	[66]
*33 Caesalpina volkensii* (Harms.) (Caesalpiniaceae)	Kenya	Omuchera (ajua) (Lh,K)	Leaves, root	Decoction	Oral	[67]
*34 Calotropis procera* (Aiton) Dryand. (Asclepiadaceae)	Benin	Wangatchiman (NS)	Leaves	Maceration	Ocular	[68]
	Mali	Fogofoko (Ba)	Leaves	Crushed	Oral, topical	[69]
*35 Cannabis sativa* L. (Cannabaceae)	South Africa	Not reported	Leaves	Not reported	Not reported	[70]
*36 Carica papaya* L. (Caricaceae)	Madagascar	Paza (NS)	Leaves, fruit, seed, roots	Not reported	Not reported	[13]
	Madagascar	Mapaza (NS)	Leaves	Not reported	Not reported	[65]
*37 Carissa edulis* (Forssk.) Vahl (Apocynaceae)	Uganda	Acuga (Lo)	Root	Maceration	Oral	[71]
	Kenya	Mukawa (NS)	Root, bark	Not reported	Not reported	[72]
*38 Cassia occidentalis* L. (*Senna occidentalis* (L.) Link) (Fabaceae)	Cote d’Ivoire	Not reported	Leaves	Crushed	Nasal	[73]
*39 Chenopodium ambrosioides* L. (Chenopodiaceae)	Uganda	Not reported	Leaves	Crushed	Nasal (inhaled)	[74]
	Uganda	Not reported	Root, leaves, flower	Not reported	Not reported	[75]
*40 Chromolaena odorata* (L.) R.M. King and H.Rob. (Asteraceae)	Nigeria	Ewe-awolowo (NS)	Leaves	Not reported	Not reported	[76]
*41 Cinnamosma fragrans* Baill. (Canellaceae)	Madagascar	Kanely (NS)	Bark	Not reported	Not reported	[13]
*42 Cissampelos fricana* A.Rich. (Menispermaceae)	Angola	Cacapa, Chitangila, Nofungi (NS)	Root	Decoction	Oral	[77]
*43 Clematis viridiflora* Bertol. (Ranunculaceae)	Mozambique	Mucoca (CT,CN)	Leaves, root	Not prepared	Nasal (inhaled)	[59]
*44 Cleome gynandra* L. (Cleomaceae)	Sudan	Tamalaika (NS)	Leaves	Decoction	Not reported	[54]
	Uganda	Not reported	Leaves	Crushed	Topical	[74]
*45 Cocculus pendulus* (J.R.Forst. and G.Forst.)Diels (Menispermaceae)	Djibouti	Cayyukto (NS)	Leaves	Burnt	Nasal (inhaled)	[78]
*46 Coffea arabica* L. (Rubiaceae)	Ethiopia	Not reported	Leaves, seed	Decoction	Not reported	[79]
*47 Combretum hereroense* Schinz (Combraetaceae)	Botswana	Mokabi (NS)	Fruit	Decoction	Not reported	[80]
*48 Commelina benghalensis* L. (Commelinacea)	Cameroon	Keyoum (bikom) (NS) Nkoleke (Bakossi) (NS)	Whole plant	Decoction	Not reported	[43]
*49 Conyza bonariensis* (L.) Cronquist (Asteraceae)	Kenya	Saruryandet (NS)	Root	Crushed	Not reported	[81]
*50 Conyza floribunda* Kunth syno (Asteraceae)	Uganda	Kafumbe (Ld)	Leaves	Decoction	Oral	[82]
*51 Crossopteryx febrifuga* (Afzel. Ex G.Don) Benth. (Rubiaceae)	Benin	Lapekoe (NS)	Bark	Burnt	Nasal (inhaled)	[83]
*52 Cyperus rotundus* L. (Cyperaceae)	Sudan	Siada (NS)	Corm	Infusion	Not reported	[54]
*53 Daniellia oliveri* (Rolfe) Hutch. and Dalziel (Fabaceae)	Mali	Sanan (Ba)	Leaves	Decoction	Oral	[69]
*54 Dicerocaryum eriocarpum* (Decne.) Abels (Pedaliaceae)	South Africa	Dinda (NS)	Whole plant	Burnt	Not reported	[37]
*55 Dichrostachys cinerea* (L.) Wight and Arn. (Fabaceae)	Nigeria	Dundu (H)	Leaves	Ointment	Topical	[84]
*56 Dysphania ambrosioides* (L.) Mosyakin and Clemants (Amaranthaceae)	Morocco	Moulbina (Th)	Whole plant	Decoction	Not reported	[85]
*57 Eucalyptus citriodora* Hook. (Myrtaceae)	Madagascar	Kininimpotsy (NS)	Leaves	Not reported	Not reported	[86]
*58 Euclea divinorum* Hiern. (Ebenaceae)	South Africa	Mutangule (V)	Leaves	Not reported	Not reported	[87]
*59 Flueggea virosa* (Roxb.ex Willd.) Royle (Phyllanthaceae)	Benin	Ichilimu (NS)	Bark	Powder	Topical	[83]
*60 Griffonia simplicifolia* (DC.) Baill. (Fabaceae)	Ghana	Not reported	Leaves	Not reported	Not reported	[12]
*61 Halocnemum strobilaceum* (Pall.) M. Bieb. (Chenopodiaceae)	Algeria	Grina (NS)	Aerial part	Not reported	Not reported	[88]
62 *Helichrysum cymosum* D.Don (Asteraceae)	South Africa	Impepho (X)	Leaves	Decoction	Not reported	[89]
*63 Helichrysum ordoratissimum* var. *ordoratissimum* (Asteraceae)	South Africa	Impepho (X)	Leaves, stem	Infusion, burnt	Oral, nasal (inhaled)	[35]
*64 Heteromorpha arborescens* (Spreng.) Cham. and Schltdl. (Apiaceae)	South Africa	Umbangandlala (X)	Leaves, root	Decoction	Oral	[90]
*65 Hypoxis hemerocallidea* Fisch, C.A.Mey and Ave’- Lall. (Hypoxidaceae)	South Africa	Ilabatheka (X)	Root	Infusion	Topical	[35]
*66 Jacaranda mimosifolia* D.Don (Bignoniaceae)	Madagascar	Zaharandaha (NS)	Leaves	Not reported	Not reported	[86]
*67 Jatropha curcas* Linn. (Euphorbiaceae)	Nigeria	Not reported	Leaves, root	Crushed	Oral	[45]
*68 Justicia schimperiana* (Hochst. Ex Nees) T. Anderson (Acanthaceae)	Ethiopia	Not reported	Leaves	Not reported	Oral	[91]
	Ethiopia	Tumuniga (Ha)	Leaves	Not reported	Oral	[92]
*69 Kigelia africana* (Lam.) Benth. (Bignoniaceae)	Uganda	Yago (Ac, Lg)	Root leaves flower	Not reported	Not reported	[93]
*70 Kniphofia caulescens* Baker (Asphodelaceae)	Lesotho	Leloele (So)	Root, bulb	Crushed	Not reported	[94]
*71 Lannea Schweinfurthii* Engl. (Anacardiaceae)	Kenya	Kwogo (NS)	Bark	Not reported	Not reported	[95]
*72 Lantana camara* L. (Verbenaceae)	Kenya	Obori bw’enyoni (NS)	Leaves	Decoction	Oral	[96]
*73 Lavandula multifida* L. (Lamiaceae)	Libya	Al-kuzami (NS)	Not reported	Not reported	Not reported	[97]
*74 Lavandula* spp (Lamiaceae)	South Africa	Lavender (X)	Leaves	Paste	Topical	[35]
*75 Leonotis leonorus* (L.) R.Br. (Lamiaceae)	South Africa	Imfincamfincane (X)	Leaves	Not reported	Nasal	[89]
*76 Lepidium sativum* L. (Brassicaceae)	Ethiopia	Feaxxo (AO)	Seed	Decoction	Oral	[58]
*77 Leucas calostachys* Oliv. (Lamiaceae)	Kenya	Moetit (NS)	Leaves	Crushed	Not reported	[81]
*78 Manihot esculenta* Crantz. (Euphorbiaceae)	Benin	Finyin (NS)	Leaves	Not reported	Not reported	[98]
*79 Marrubium vulgare* L. (Lamiaceae)	Algeria	Temeriouit (NS)	Aerial part	Decoction	Not reported	[99]
*80 Mentha longifolia* (L.) L. (Lamiaceae)	South Africa	Ballerja (A)	Leaves	Crushed	Topical	[100]
	South Africa	Bullerja (A)	Stem, leaves	Decoction	Not reported	[101]
	South Africa	Ballerja (A)	Leaves	Crushed	Topical	[100]
*81 Microglossa pyrifolia* (Lam.) Kuntze (Asteraceae)	Tanzania	Omuhe/Mkuraiju (NS)	Leaves	Crushed	Nasal	[102]
*82 Milicia excelsaexcels* (Melw.) C. C. Berg (Moraceae)	Ghana	Not reported	Bark	Not reported	Not reported	[12]
*83 Momordica charantia* L. (Cucurbitaceae)	Benin	Nyensiken (NS)	Whole plant	Decoction	Oral	[68]
*84 Momordica foetida* Schumach. (Cucurbitaceae)	Ethiopia	Achcha (Hd)	Leaves	Not reported	Not reported	[103]
*85 Moringa oleifera* Lam. (Moringaceae)	Benin	Kpatiman/Kpatima vovo (NS)	Leaves	Maceration	Ocular	[68]
	Tanzania	Mlonge (Sw, Ku)	Leaves, bark, root	Decoction, infusion	Oral	[104]
*86 Nicotiana tabacum* L. (Solanaceae)	Cameroon	Tobacco,ndabah (bamileke) (NS)	Leaves	Plaster	Not reported	[43]
*87 Nigella sativa* L. (Ranunculaceae)	Sudan	Kamoon aswad (NS)	Seed	Infusion	Not reported	[54]
	Nigeria	Tiqur azmud (NS)	Seed	Crushed	Nasal (sniffed)	[47]
	Sudan	Kamoon aswad (NS)	Seed	Infusion	Not reported	[54]
*88 Nymphaea caerulea *Savigny (*Nymphaea nouchali* var. *caerulea* (Savigny) Verdc.) (Nymphaeaceae)	Egypt	Kurunb el ma (NS), Bashnin arabi (NS)	Root	Maceration	Not reported	[105]
*89 Ocimum basilicum* L. (Lamiaceae)	South Africa	Not reported	Leaves	Infusion	Oral	[42]
	Uganda	Emopim (At, Lu)	Leaves	Not reported	Not reported	[106]
*90 Ocimum gratissimum* L. (Lamiaceae)	Cote D’ivoire	Alomagninrin (NS)	Leaves	Decoction, pilage	Nasal, oral	[41]
	Madagascar	Romba be (NS)	Leaves	Not reported	Not reported	[13]
	Cameroon	Kaja (NS)	Leaves	Not reported	Nasal	[107]
	Ethiopia	Damakasiya malaa (NS)	Leaves	Crushed	Oral, nasal	[56]
	Cote D’ivoire	Not reported	Leaves	Crushed	Nasal	[73]
	Cote D’ivoire	Alomagninrin (NS)	Leaves	Decoction	Nasal, oral	[41]
	Ghana	Nunum (T)	Leaves, seed	Decoction	Oral	[51]
*91 Ocimum lamiifolium* Hochst. Ex Benth (Lamiaceae)	Nigeria	Damakesse (NS)	Leaves	Crushed	Oral, nasal (sniffed)	[47]
*92 Ocotea bullata * (Burch.) E. Meyer in Drege (Lauraceae)	South Africa	UmNukani (Z)	Bark	Not reported	Not reported	[57]
*93 Periploca linearifolia* Quart. Dill. and A. Rich. (Euphorbiaceae)	Kenya	Sinendet (NS)	Root, leaves	Decoction	Not reported	[81]
*94 Phyllarthron bojeranum* DC. (Bignoniaceae)	Madagascar	Zahana (NS)	Leaves	Not reported	Not reported	[86]
*95 Bauhinia reticulata* DC (Fabaceae)	Cameroon	Not reported	Root	Not reported	Not reported	[108]
*96 Greenwayodendron suaveolens* (Engl. & Diels) Verdc.) (Annonaceae)	Cameroon	Pygmies Bakola (NS)	Stem, bark	Decoction	Oral	[109]
	Nigeria	Not reported	Bark	Crushed	Topical	[66]
*97 Prosopis Africana* (Guill. and Perr.) Taub. (Mimosaceae)	Nigeria	Sanchi (N)	Stem, bark	Infusion	Not reported	[107]
*98 Psidium guajava* L. (Myrtaceae)	Nigeria	Goba (H)	Leaves	Not reported	Not reported	[110]
*99 Ptaeroxylon obliquum* (Thunb.) Radlk. (Rutaceae)	South Africa	Umthathe (Z)	Bark	Not reported	Not reported	[52]
*100 Rapanea melanophloeos* (L.) Mez (Myrsinaceae)	South Africa	Itshongwe (X), umaphiph (X)	Leaves	Infusion, decoction	Oral	[35]
*101 Ricinus communis* L. (Euphorbiaceae)	Morocco	Thazartoqzine (Th)	Leaves	Infusion	Not reported	[85]
	South Africa	Umhlamvuthwa (X)	Leaves, root	Not reported	Not reported	[111]
	Uganda	Mukakaala (NS)	Leaves	Crushed	Not reported	[40]
*102 Ruta chalepensis* L. (Rutaceae)	Ethiopia	Xenaaddaa (NS)	Leaves	Not reported	Not reported	[112]
*103 Ruta graveolens* L. (Rutaceae)	South Africa	Not reported	Leaves	Decoction	Oral	[42]
*104 Salvia aegyptiaca* L. (Lamiaceae)	Libya	Tefah El-Shah (NS)	Aerial part	Not reported	Not reported	[97]
*105 Salvia africana-caerulea* L. (Lamiaceae)	South Africa	Blousalie/bloublom (A)	Leaves	Decoction	Not reported	[101]
*106 Securidaca longipedunculata* Fresen. (Polygalaceae)	Nigeria	Jechi (N)	Root, bark	Powder	Nasal (inhaled)	[107]
*107 Sida acuta* Burm.f. (Malvaceae)	Gabon	Not reported	Leaves	Decoction	Topical	[113]
*108 Solanum incanum* L. (Solanaceae)	Botswana	Tholwana-e-tona (Se)	Root	Decoction	Oral	[114]
	Kenya	Entulelei (Ma)	Root	Not reported	Not reported	[44]
	Ethiopia	Abkewa (G)	Root, leaves	Chewed	Oral	[49]
*109 Strophanthus hispidus* DC. (Apocynaceae)	Ghana	Edupeyi (T)	Leaves	Decoction	Oral	[115]
*110 Strychnos decussata* (Pappe) Gilg (Loganiaceae)	South Africa	Muvhavhanyane (V)	Bark	Not reported	Not reported	[87]
*111 Vepris nobilis* (Delile) Mziray) (Rutaceae)	Kenya	Kuryot (NS)	Root	Decoction	Not reported	[116]
*112 Tulbaghia acutiloba* Harv. (Alliaceae)	South Africa	Isivumbampuzi (X)	Root, leaves	Decoction	Oral	[35]
*113 Vernonia amygdalina* Delile (Asteraceae)	Uganda	Mululuza (Ld)	Root, leaves	Decoction	Oral	[82]
	Uganda	Not reported	Root	Crushed	Oral	[74]
*114 Warburgia ugandensis* Sprague (Canellaceae)	Kenya	Sokwon (NS)	Bark	Burnt	Nasal (sniff)	[117]
*115 Xylopia staudtii* Engl. and Diels (Annonaceae)	Nigeria	Not reported	Bark	Powder	Oral	[107]
*116 Xysmalobium undulatum* (L.) W.T. Aiton (Apocynaceae)	South Africa	Itshongwe (X)	Root, leaves	Powder	Nasal (sniffed)	[90]
*117 Zea mays* L. (Poaceae)	South Africa	Not reported	Root	Not reported	Not reported	[70]

# Local names of plant species (known in a language specific to a particular geographical area of study in the various countries): Afrikaans (A), Acholi (Ac), Amharic (Am), Afan Oromo (AO), Arafe (Ar), Ateso (At), Baka (Ba), Chadian Arabic (CA), ChiNdau (CN), ChiTewe (CT), Gumz (G), Hausa (H), Hadiyigna (Hd), Kamba(Ka), Kiswahili (Ki), Kiluguru (Ku), Luganda (Ld), Langi (Lg), Luhya (Lh), Lango (Lo), Lugwere (Lu), Maasai(Ma), Moore (Mo), Nupe (N), Not stated (NS), Sepedi (S), Setswana (Se), Sotho (So), Swahili (Sw), Twi (T), Tharafit (Th), Venda (V), Xhosa (X), Xitsonga (Xi), Yoruba (Y), Zulu (Z).

## Data Availability

All data used for the review are included in the manuscript.
